# *Phyllanthus* Suppresses Prostate Cancer Cell, PC-3, Proliferation and Induces Apoptosis through Multiple Signalling Pathways (MAPKs, PI3K/Akt, NF**κ**B, and Hypoxia)

**DOI:** 10.1155/2013/609581

**Published:** 2013-04-16

**Authors:** Yin-Quan Tang, InduBala Jaganath, Rishya Manikam, Shamala Devi Sekaran

**Affiliations:** ^1^Department of Medical Microbiology, Faculty of Medicine, University of Malaya, 50603 Kuala Lumpur, Malaysia; ^2^Biotechnology Centre, Malaysia Agricultural Research and Development Institute (MARDI), 43400 Serdang, Malaysia; ^3^Department of Trauma and Emergency Medicine, University Malaya Medical Centre, 46150 Kuala Lumpur, Malaysia

## Abstract

*Phyllanthus* is a traditional medicinal plant that has been found to have antihepatitis, antibacterial, and anticancer properties. The present studies were to investigate the *in vitro* molecular mechanisms of anticancer effects of *Phyllanthus* (*P. amarus, P. niruri, P. urinaria,* and *P. watsonii*) plant extracts in human prostate adenocarcinoma. The cancer ten-pathway reporter array was performed and revealed that the expression of six pathway reporters were significantly decreased (Wnt, NF*κ*B, Myc/Max, hypoxia, MAPK/ERK, and MAPK/JNK) in PC-3 cells after treatment with *Phyllanthus* extracts. Western blot was conducted and identified several signalling molecules that were affected in the signalling pathways including pan-Ras, c-Raf, RSK, Elk1, c-Jun, JNK1/2, p38 MAPK, c-myc, DSH, *β*-catenin, Akt, HIF-1*α*, GSK3*β*, NF*κ*B p50 and p52, Bcl-2, Bax, and VEGF, in treated PC-3 cells. A proteomics-based approach, 2D gel electrophoresis, was performed, and mass spectrometry (MS/MS) results revealed that there were 72 differentially expressed proteins identified in treated PC-3 cells and were involved in tumour cell adhesion, apoptosis, glycogenesis and glycolysis, metastasis, angiogenesis, and protein synthesis and energy metabolism. Overall, these findings suggest that *Phyllanthus* can interfere with multiple signalling cascades involved in tumorigenesis and be used as a potential therapeutic candidate for treatment of cancer.

## 1. Introduction

Prostate cancer is among one of the major life-threatening cancers and is the second most frequently diagnosed cancer after lung cancer in men. Even with advances in early detection and conventional treatment strategies, prostate cancer incidence has increased worldwide, and it has become more resistant to available treatments. Generally, tumour metastasis confers poor prognosis and remains a major obstacle in the inability to improve patient outcome, where at least 50% of prostate cancer patients are diagnosed with pathologic or clinical evidence of bone metastasis [1, 2]. Bones metastases can impair the quality of life and reduce life expectancy with its wide range of symptoms and complications including severe pain, pathologic fractures, and epidural spinal cord compression. Osteolytic metastases can also result in life-threatening hypercalcemia [3, 4]. Hence, there is an urgent need of new anticancer agents which can combat both local and metastatic prostate cancer.

During the tumour progression, cancer cells acquire several distinct characteristic alterations from normal cells which include the capacities to proliferate independently of growth-promoting or growth-inhibitory signals, the ability to undergo metastasis and angiogenesis, and to evade apoptosis [5].

Cellular signalling pathways are always interconnected to form complex signalling networks. These interactions are important for a cell to regulate vital and diverse processes such as protein synthesis, cell growth, immune responses, differentiation, and homeostasis and cell death [6]. These interactions can be achieved either directly by cell-cell contact or indirectly by intracellular signalling molecules. However, when this intercellular communication is disrupted either by external (e.g., UV radiation and chemicals) or internal factors (e.g., hereditary mutation), diseases such as cancer, diabetes, and developmental defects can occur. Most of these signalling proteins have become anticancer targets by induction of apoptosis and/or inhibition of tumour metastasis and angiogenesis [7]. Thus, understanding these underlying (molecular, biochemical, cellular, and physiological) mechanisms in cancer is of paramount importance.

The actual molecular events leading to prostate metastasis remain elusive. The mitogen-activated protein kinase family members (MAPKs) are believed to play a part in mediating metastasis by inducing proteolytic enzymes that lead to degradation of basement membrane and those that promote cell migration and activate pro-survival genes and cell growth [7]. MAPK pathway has been found to be highly expressed in various cancers such as breast, colon, melanoma, prostate, and lung, suggesting a role for MAPK pathway in tumour progression and metastasis [9–12]. The MAPK pathway is activated by different extracellular stimuli and has distinct downstream targets, hence a disruption on MAPK pathway could halt cancer progression by inhibition of tumour angiogenesis, proliferation, invasion, and metastasis [7]. In prostate cancer, several other pathways have also been shown to be highly expressed and these include PI3 K/Akt, NF*κ*B, and Wnt signal transduction pathways. The expressions of these pathways have been associated with tumour development and progression [13–15].

During tumour metastasis, the hypoxia-inducible factors (HIFs) which regulate oxygen delivery and consumption are highly expressed. Activated HIFs will induce transcription of survival-related genes to produce vascular endothelial growth factor (VEGF) and stimulate tumour angiogenesis and thereby increase oxygen transport [16]. These regulations have caused hypoxic cancer cells to acquire invasive and metastatic properties as well as to develop resistance to chemotherapy.

In this study, we attempted to investigate the underlying molecular mechanism of *Phyllanthus *against prostate adenocarcinoma. *Phyllanthus, *of the family Euphorbiaceae, is a widely distributed plant and reported to have many pharmacological effects such as antiviral [17, 18], antibacterial, [19] and antihepatotoxic [20] as well as anticancer properties [21].

We have previously reported four species of *Phyllanthus* to have antiproliferative effect and capability to induce apoptosis in human prostate carcinoma cells [8], and currently we delved further into understanding the molecular mechanism and intracellular signalling networks changes behind these properties of *Phyllanthus*. We also seek to identify possible roles of *Phyllanthus *as a new target for therapeutic intervention and pharmacological manipulation in prostate cancer research.

## 2. Materials and Methods

### 2.1. Preparation of *Phyllanthus* Extracts

Four different species of *Phyllanthus* (*P. amarus, P. niruri, P. urinaria, *and *P. watsonii*) were used in this study. For each of these species, two extracts were prepared: aqueous and methanolic. The preparation of *Phyllanthus* extracts was prepared as described previously by Tang et al. [8]. Briefly, fresh plant samples were harvested, washed, and freeze-dried. Ultrapure water was used to soak the dried plant samples for aqueous extract preparation, while absolute methanol was used to prepare the methanolic extract. Then, the samples were subjected to homogenization with extraction buffer, and the supernatant was obtained after three rounds of extraction. Lyophilized forms of aqueous and methanolic extracts were collected after evaporation and stored at −20°C prior to experimentation.

### 2.2. Cell Culture

Human prostate adenocarcinoma cancer cell, PC-3, was obtained from American Type Culture Collection (Rockville, MD). Cells were cultured with RPMI-1640 (Roswell Park Memorial Institute) and supplemented with 10% heat-inactivated fetal bovine serum (FBS, Gibco). Cells were maintained in culture at 37°C in a humidified atmosphere of 5% CO_2_ and 95% humidity.

### 2.3. Cignal Finder 10-Pathway Reporter Arrays Analysis

#### 2.3.1. Dual Luciferase Pathway Reporter Transient Transfection

Ten different cancer-related pathways analysis was performed using the Cignal Finder 10-Pathway Reporter Arrays (SA Biosciences, Fredrick, MD) according to the manufacturer's instructions. Optimization of the conditions, including amount and incubation time of plasmid construct of transcription factor-responsive reporter of each pathway into the cells, was performed to ensure high transfection efficiency and inhibit transformation. Reverse transfection protocol was implemented for this assay. Prior to experiment, PC-3 cells were seeded into a 96-well white plate and incubated overnight at 37°C. One hundred nanograms of plasmid construct of transcription factor-responsive reporter of each pathway and controls were added to cells and incubated overnight in a 37°C incubator with 5% CO_2_. After 24 h of transfection, the old media was discarded and the cells were treated with *Phyllanthus* extracts at their respective IC_50_ values (Table 1) for another 24 h. Each transfection condition was carried out in triplicates.

#### 2.3.2. Luciferase Assay

Each of the pathways/reporters consists of an inducible transcription factor-responsive firefly luciferase reporter and the constitutively expressing *Renilla* construct. *Renilla* construct is to act as an internal control for normalizing transfection efficiencies and monitoring cell viability. After 24 h, the activity of each pathway was determined by measuring the generated firefly and *Renilla *luminescent signals using the Dual-Glo Luciferase Assay system (Promega, Madison, WI) on the Glomax machine (Promega, USA). The relative luciferase units were determined by dividing the firefly to *Renilla* luciferase activity ratio.

### 2.4. Western Blot Analysis

The intracellular signalling molecules in MAPK (pan-Ras, c-Raf, Elk, Rsk, c-Jun, JNK, Akt, and p38 MAPK), Wnt (GSK3*β*, *β*-catenin, and DSH), Myc/Max (c-Myc), and Hypoxia (c-myc, HIF-1*α*, and VEGF) and NF*κ*B (p50 and p52) pathways as well as apoptotic (Bcl-2 and Bax) proteins were chosen for western blot analysis to determine their expression upon *Phyllanthus* treatment in PC-3. All these antibodies were purchased from Merck (USA). Briefly, protein lysates were resolved on 12% SDS-PAGE gels. After electrophoresis, proteins were transferred onto nitrocellulose membranes. The membranes were blocked and incubated with primary antibodies overnight at 4°C. Subsequently, the membranes were washed with TBST and incubated with appropriate secondary antibodies (horseradish-conjugated goat antimouse or antigoat IgG) for 1 h. ImageJ software was used to measure the band intensity. The percentage of protein expression was calculated by dividing the band intensity of treated group with untreated group.

### 2.5. 2D Gel-Based Proteomics Analysis

#### 2.5.1. Two-Dimensional Gel Electrophoresis

A total of 500 mg total protein was subjected to 2D gel electrophoresis according to the manufacturer's instructions (GE Healthcare). Briefly, total proteins were extracted from untreated and treated groups by incubation with lysis buffer on ice for 30 minutes. The protein pellets were resolubilized in rehydration solution (8 M urea, 2% CHAPS, 40 mM DTT, 0.5% IPG buffer pH3-11NL, bromophenol blue) and kept at −80°C until further analysis. Total amount of proteins was determined using 2D Quant kit (GE Healthcare), and 500 mg of proteins were rehydrated into 13 cm immobilized pH gradient (IPG) strips (pH 3–11 nonlinear) (GE Healthcare). The first dimension was run on the IPGphor III machine (GE Healthcare) at 20°C with the following settings: step 1 at 500 V for 1 h; step 2 at 500–1000 V for 1 h; step 3 at 1000–8000 V for 2.5 h; and step 4 at 8000 V for 0.5 h. Upon completion of first-dimensional separation, the strip was equilibrated as following: first reduction with 64.8 mM of dithiothreitol (DTT)-SDS equilibration buffer (50 mMTris-HCl (pH 8.8), 6 M urea, 30% glycerol, 2% SDS, 0.002% bromophenol blue) for 15 minutes, followed by alkylation with 135.2 mM of iodoacetamide (IAA)-SDS equilibration buffer for another 15 minutes. The second-dimension electrophoresis was performed by electrophoresing the samples in 12.5% SDS acrylamide gels by using the SE600 Ruby system (GE Healthcare) at 25°C in an electrode buffer (25 mMTris, 192 mM glycine, and 0.1% [wt/vol] SDS) with the following settings: step 1 at 100 V/gel for 45 minutes; and step 2 at 300 V/gel until the run is completed. After electrophoresis, the gels were fixed with destaining solution for 30 minutes, followed by staining with hot Coomassie blue for 30 minutes. Lastly, the gels were scanned using Ettan DIGE Imager (GE Healthcare). Gel images were analysed using PDQuest 2-D Analysis Software (Bio-Rad, USA), and only protein spots which showed significant differences (more than 1.0 fold) were selected for mass spectrometry analysis.

#### 2.5.2. Protein Digestion, Desalting, and MALDI-TOF/TOF Analysis

The significant protein spots were manually excised from the polyacrylamide gels and kept in sterile 1.5 mL Eppendorf tubes. Excised spots (gel plugs) were washed with destaining solution (50 mM NH_4_HCO_3_) until the gel plugs were clear. The gel plugs were then incubated with reducing solution (100 mM NH_4_HCO_3_ containing 10 mM DTT) for 30 min at 60°C. Then, the plugs were alkylated with 100 mM NH_4_HCO_3_ containing 55 mM IAA for 20 min in the dark and followed with three times of 50% acetone in 100 mM NH_4_HCO_3_ for 20 min each. The gel plugs were then rehydrated with 100% ACN. In-gel digestion using trypsin gold (Promega, Mass Spectrometry Grade) was added into gel plug and incubated overnight at 37°C. Proteins were extracted from gel plugs and were purified using Ziptip (Ziptip C18, Millipore, Bedford, MA, USA). The eluted proteins were mixed with MATRIX solution and spotted on MALDI plate using dry droplet method and analysed using AbSciexTof/Tof instruments. The generated peptides were blasted with MASCOT search algorithm (version 2.1.0) to identify the possible proteins.

### 2.6. Statistical Analysis

For all experiments, results were expressed as the mean ± standard error (SEM) of data obtained from triplicate experiments using SPSS software. The Student's *t*-test was used where values of *P* < 0.05 were considered significant.

## 3. Results

### 3.1. *Phyllanthus* Alters Expression of Several Cancer Pathways

**Figure 1:**
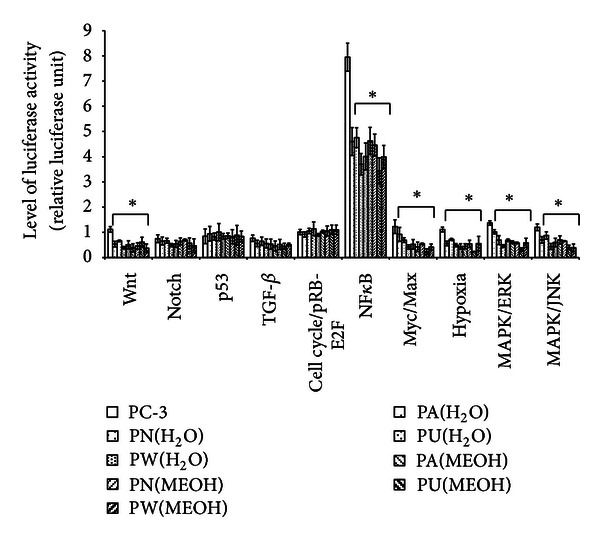
The expression of transcription activities in ten-cancer related pathways of untreated and treated PC-3. Only six different pathways (Wnt, NF*κ*B, Myc/Max, Hypoxia, MAPK/ERK, and MAPK/JNK) were significantly downregulated (*P* < 0.05), and for the others no significant changes were observed. Bars show the mean percentage ± SEM. *P ≤ 0.05 and **P ≤ 0.01 versus control.

Ten different cancer-related pathways were investigated including Wnt, Notch, p53, TGF-*β*, cell cycle/pRB-E2F, NF*κ*B, Myc/Max, Hypoxia, MAPK/ERK, and MAPK/JNK pathways. The differential expression of each of these pathways is presented in Figure 1. From Figure 1, it was distinguished that in the untreated PC-3 cells, all ten investigated pathways were expressed to regulate the cells growth and survival. However, the *Phyllanthus*-treated cells showed a significant down-regulation of six pathways: Wnt, NF*κ*B, Myc/Max, Hypoxia, MAPK/ERK, and MAPK/JNK, suggesting that the plant exerts its properties by targeting these pathways (*P* < 0.05). The other pathways were not significantly affected by *Phyllanthus* extracts (*P* > 0.05).

### 3.2. *Phyllanthus* Disrupts Antiapoptotic/Proapoptotic Balance

**Figure 2:**
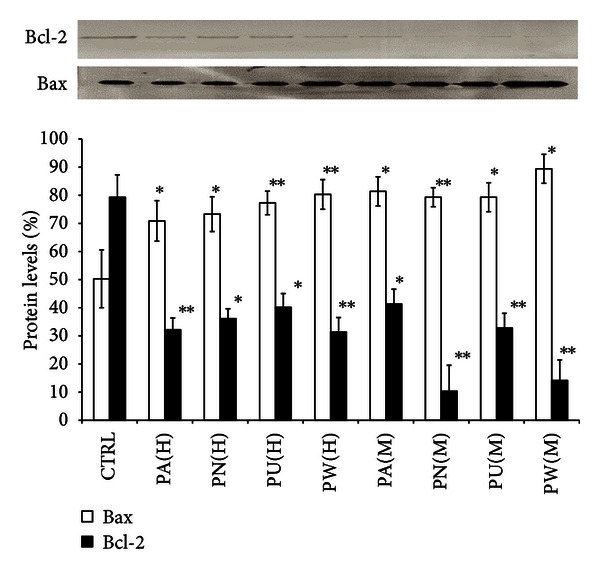
Disruption of *Phyllanthus* on antiapoptotic/proapoptotic balance in PC-3. The expression of proapoptotic (Bax) was upregulated and antiapoptotic (Bcl-2) protein was downregulated after treatment with *Phyllanthus* extracts. Bars show the mean percentage ± SEM. *P ≤ 0.05 and **P ≤ 0.01 versus control.

One of the hallmarks of cancer is the inhibition of apoptosis. This can be achieved by suppressing the expression of proapoptotic protein, Bax, and stimulating the expression of antiapoptotic protein, Bcl-2, as shown in Figure 2. The graph shows a significant increase of Bax protein in the *Phyllanthus*-treated cells (*P* < 0.05) with a concurrent decrease in Bcl-2 (*P* < 0.05) as compared to the untreated cells. Among the species of *Phyllanthus, *both aqueous and methanolic extracts of *P. watsonnii* showed the most significant changes on Bax and Bcl-2 expression (*P* < 0.01), followed by *P. urinaria, P. niruri,* and *P. amarus*.

### 3.3. *Phyllanthus* Alters MAPKs Signalling Activities in Treated PC-3 Cells

**Figure 3:**
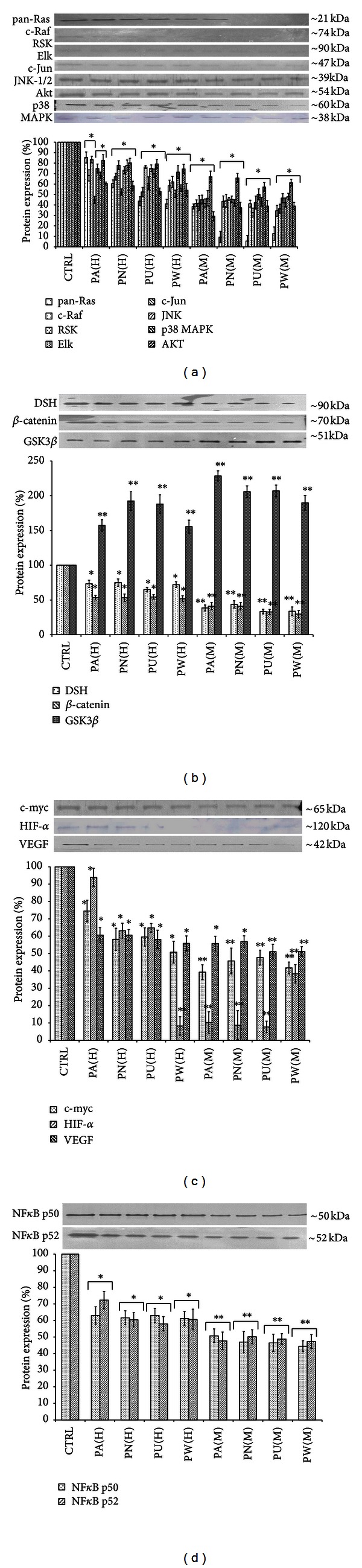
Inhibition of cellular signalling pathways in PC-3 cells by *Phyllanthus* extracts. (a) Suppression of MAPKs and PI3 K/Akt pathways via inhibition on Ras, Raf, Elk, Rsk, Jnk, c-Jun, p38 MAPK, and Akt proteins. (b) Inhibition of Wnt pathway via down-regulation of dishevelled (DSH) and *β*-catenin proteins in conjunction with upregulation of GSK3*β*. (c) Inhibition of Myc/Max and hypoxia pathways via down-regulation of c-myc, HIF-1*α*, and VEGF. (d) Inhibition of NF*κ*B pathway was observed when the expressions of p50 and p52 proteins were significantly downregulated. Bars show the mean percentage ± SEM.*P ≤ 0.05 and **P ≤ 0.01 versus control.

Two upstream activators, pan-Ras and c-Raf, were highly expressed in untreated PC-3 to ensure constitutive activation of MAPK pathways (Figure 3(a)). Another upstream activator of Akt was also found to be highly expressed in PC-3 cells. The constitutive activations of pan-Ras, c-Raf, and Akt can activate their downstream targets in three different MAPK pathways: MAPK/ERK (RSK, Elk1, c-Jun/AP-1), MAPK/JNK (JNK1/2), and p38 (p38 MAPK). As shown in Figure 3(a), the detected expressions of all these intracellular signalling molecules in untreated PC-3 cells indicate their involvement in regulating PC-3 cells' growth. Nevertheless, all these signalling molecules proteins were notably downregulated in PC-3 cells treated with different species of *Phyllanthus* (*P* < 0.05).

### 3.4. *Phyllanthus* Alters Wnt, Myc/Max, and Hypoxia Signalling Activities in Treated PC-3 Cells

The expression of Wnt signalling pathway was detected at increased levels in PC-3 cells (Figure 1) but was downregulated when treated with *Phyllanthus*. Investigation of the downstream molecules in this pathway revealed expression of three intracellular signalling molecules: dishevelled (DSH) at 95 kDa, glycogen synthase kinase 3-beta (Gsk3*β*) at 47 kDa, and *β*-catenin at 65 kDa. After treatment with aqueous (*P* < 0.05) and methanolic (*P* < 0.01) extracts of *Phyllanthus* species as shown in Figure 3(b), the expression of DSH and *β*-catenin was noted to be significantly downregulated. Contrarily, the expression of Gsk3*β* was significantly upregulated in treated PC-3 cells as compared to untreated PC-3 cells (*P* < 0.01). In Myc/Max and hypoxia pathways, three different intracellular signalling molecules were detected by western blot. As shown in Figure 3(c), the expression of the c-myc was renowned to be downregulated in aqueous- (*P* < 0.05) and methanolic-treated (*P* < 0.01) PC-3 cells. The downstream targets of c-myc: the expression of HIF-1*α* and VEGF in PC-3 were significantly downregulated by *Phyllanthus *extracts (*P* < 0.05).

### 3.5. *Phyllanthus* Alters NF*κ*B Signalling Pathway in Treated PC-3 Cells

Two members of NF*κ*B signalling pathway were detected in PC-3 cells: NF*κ*Bp50 and NF*κ*Bp52. As shown in Figure 3(d), both proteins were significantly downregulated in PC-3 after treatment with aqueous (*P* < 0.05) and methanolic (*P* < 0.01) extracts of *Phyllanthus *species as compared to untreated cells. Among the *Phyllanthus* species, *P. urinaria* showed the strongest inhibitory effects on these intracellular signalling molecules in affected pathways, followed by *P. watsonii, P. amarus,* and *P. niruri* for both aqueous and methanolic extracts.

### 3.6. Proteomic Profiling of the Differentially Expressed Proteins in *Phyllanthus* Treated PC-3 Cells

**Figure 4:**
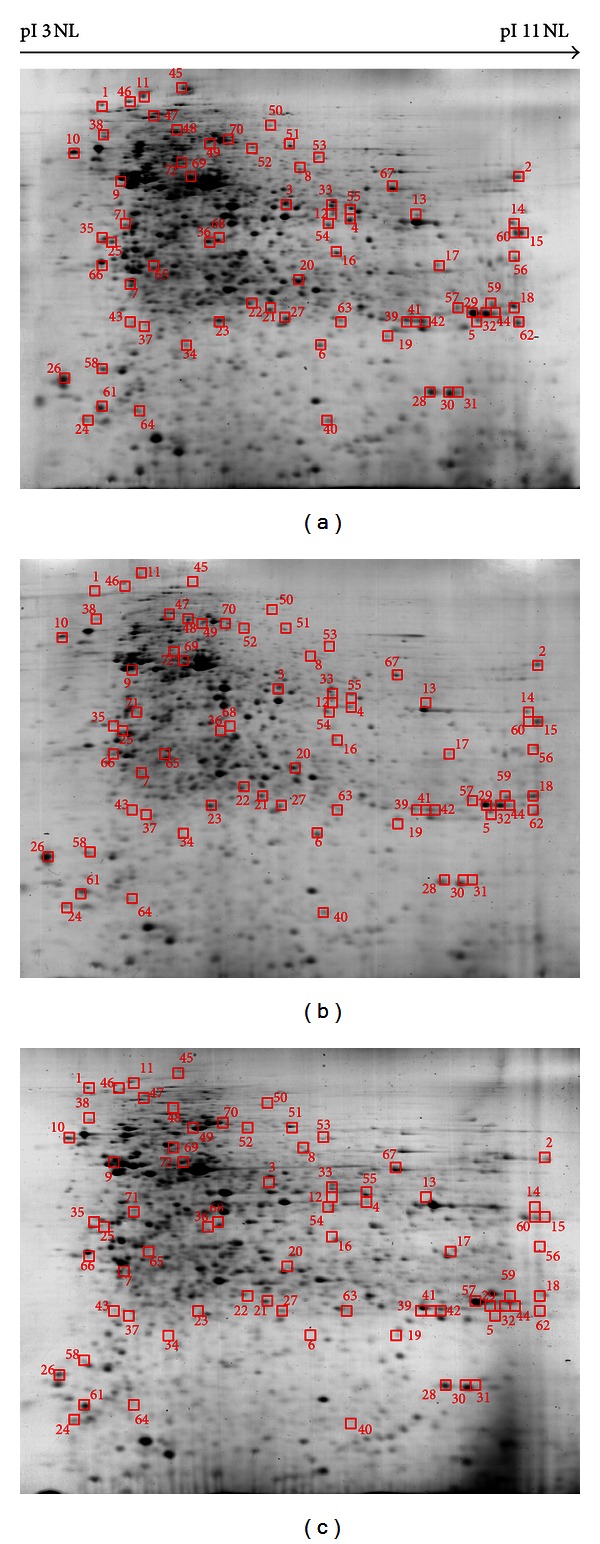
Proteomic profiles of *Phyllanthus*-untreated and treated PC-3 cells. The highlighted spots were differentially expressed proteins in (a) untreated PC-3 cells, compared to (b) aqueous- and (c) methanolic-treated PC-3 cells.

Differentially expressed proteins were statistically defined based on two criteria: (1) degree of intensity >1.0 fold (protein scores of greater than 70 are considered significant, *P* < 0.05) and (2) reoccurrence of the same proteins in the three repeated experiments (Figure 4). According to these criteria, 72 proteins were identified by MS/MS and grouped into four biological processes based on their functions described in UniProtKB/Swiss-Prot protein database (Table 2).

#### 3.6.1. Regulation of Protein Involved in Actin Cytoskeleton and Metastasis

In Group I (cell adhesion, migration, invasion, and metastasis), 10 proteins were found to be differentially expressed in *Phyllanthus*-treated PC-3 cells. Of these, three proteins (keratin, type II cytoskeletal 8 and type I cytoskeletal 9, and keratin-associated protein 3-1) were observed to be upregulated. Notably, these upregulated proteins were derived from the same family, keratin, and their expressions were about 1-2 folds higher than untreated cells. The 7 other proteins found to be downregulated were Ephrin-B1, actin, EH domain-binding protein 1, heat shock protein 1, vimentin, tubulin alpha-8 chain, and MEMO1.

#### 3.6.2. Regulation of Protein Involved in Proliferation, Cell Cycle, and Apoptosis

In Group II (proliferation, cell cycle, and apoptosis), 28 proteins were significantly downregulated in treated PC-3 cells. Among these altered proteins, five, namely gluthathione S-transferase P, protein Wnt-5a, proto-oncogene Wnt-3, putative Ras-related protein Rab-42, and GTPAse HRas precursor, showed the greatest reduction in their expression with a range of 1.7–2.2 folds higher than untreated cells (*P* < 0.05).

#### 3.6.3. Regulation of Glycogenesis and Glycolysis

In Group III (glycogenesis and glycolysis), 7 downregulated proteins were identified in the treated PC-3 cells, and five of them were enzymes: phosphoglycerate kinase-1, alpha-enolase, glyceraldehyde-3-phosphate dehydrogenase (G3PD), fructose-biphosphate aldolase, and triosephosphate isomerase.

#### 3.6.4. Regulation of Protein Synthesis and Energy Metabolism

In Group IV (protein synthesis and energy metabolisms), 27 proteins were differentially expressed with only one being significantly upregulated in PC-3 after treatment with *Phyllanthus *extracts: voltage-dependent anion-selective channel protein 1 in the range of 1.5–1.8 folds higher. Among the downregulated proteins detected, four associated with calcium regulation were detected: 39S ribosomal protein L51, calumenin, calreticulin, and 78 kDa glucose-regulated protein.

## 4. Discussion

Presently, modern therapies for cancer treatment such as surgery, chemotherapy, and immunotherapy are deemed relatively unsuccessful due to their ineffectiveness and safety issues, as well as costliness [22]. Although chemotherapy was advocated at one time, recent studies have implied that these agents are no longer effective as they used to be, mainly because cancer cells are able to evoke other survival pathways, and these agents end up being cytotoxic. Hence, anticancer agents that can target multiple pathways hold more promise in the future of cancer treatment.

We have previously shown *Phyllanthus *to exhibit selective cytotoxicity on prostate cancer cells and apoptosis induction [8]. This was mainly attributed to the mixture of bioactive compounds within the *Phyllanthus* plant such as gallic acid and geraniin which are capable of inducing apoptosis. Gallic acid is known to induce apoptosis through suppression of NF*κ*B, MAPK, and PI3 K/Akt pathways, while geraniin regulates the p53 pathway [23, 24]. As no individual class of components could be fully responsible for the activity/effect produced by a whole extract [25], we found it more meaningful to assess the activity of *Phyllanthu*s extract as a whole mixture of bioactive compounds rather than as their individual compounds.

**Figure 5:**
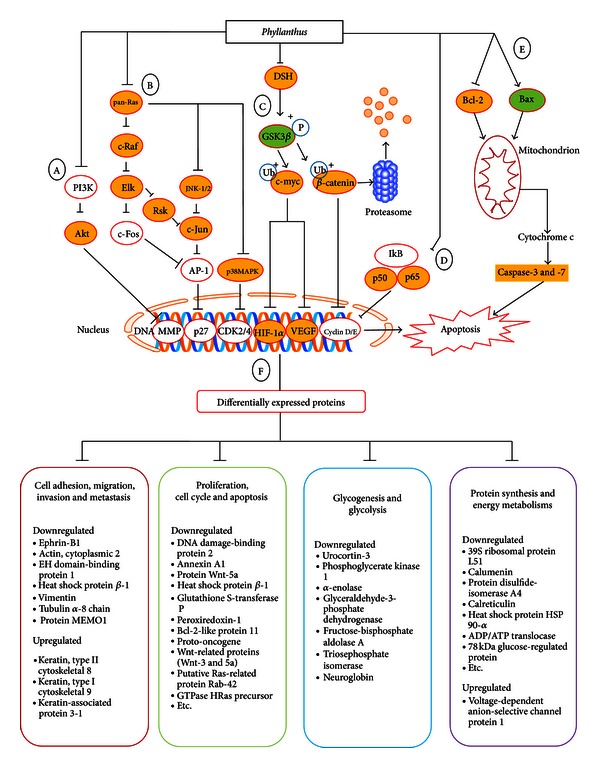
Schematic diagram illustrating that *Phyllanthus* regulates multiple signalling pathways and protein activities in PC-3 cells. The suppression of (a) PI3K/Akt (Akt protein), (b) MAPKs (pan-Ras, c-Raf, RSK, Elk1, c-Jun, JNK1/2, and p38 MAPK protein), (c) Wnt (DSH, Gsk3*β*, and *β*-catenin proteins), Myc/Max (c-myc protein), hypoxia (HIF-1*α* and VEGF proteins) and NF*κ*B (p50 and p52 proteins) pathways by *Phyllanthus* plant extracts in PC-3 cells. (e) Apoptosis induction via up-regulation of Bax protein and down-regulation of Bcl-2 to induce caspase-3/7 activation in treated PC-3 cells. (f) A number of proteins involved in proliferation, cell cycle, apoptosis, metastasis, glycogenesis and glycolysis, protein synthesis as well as energy metabolism were found altered in PC-3 cells upon *Phyllanthus* treatment.

Cancer cells survival requires the inhibition of apoptosis, which is accomplished by suppressing the expression of proapoptotic proteins (Bax) as well as promoting the expression of antiapoptotic proteins (Bcl-2) [26]. However, the expression of Bcl-2 in *Phyllanthus*-treated PC-3 cells was greatly suppressed, accompanied by the up-regulation of Bax expression (Figure 2). The highly expressed Bax protein in PC-3 after treatment with *Phyllanthus* extract could induce cytochrome *c* release from mitochondria which can then induce proteolytic activation of procaspase-9. This in turn activates caspase-3 and -7 as detected in our previous report [8] and finally leads to apoptosis induction in PC-3 cells (Figure 5).

Ras proteins are membrane-bound GTPases responsible for transmitting extracellular signals into the nucleus to regulate genes-driven malignancy of cancer including increased proliferation, apoptosis evasion, metastasis, and angiogenesis [27]. The observed down-regulation of pan-Ras proteins by the *Phyllanthus *could then lead to suppression of their downstream targets: c-Raf and Akt (Figure 3(a)). The major downstream targets of c-Raf and Akt can be subdivided into three mitogen-activated protein kinase (MAPK) pathways: ERK 1/2, JNK1/2, and p38 MAPK.

In cancer cells, the active ERK1/2 protein will activate RSK2 and Elk1 proteins that subsequently activate c-Jun and c-Fos proteins. Both c-Jun and c-Fos will then combine to form activator protein 1 (AP-1) which is a transcription factor that regulates survival genes [27]. Besides that, JNK-1/2 and p38 MAPK pathways are also involved in enhancing AP-1 formation by also producing c-Jun and c-Fos (Figure 5) [7]. However, all these intracellular signalling molecules involved in the MAPK signalling were found to be downregulated in PC-3 cells after treatment with *Phyllanthus* extracts (Figure 3(a)). This indicates that *Phyllanthus *extracts are capable of disrupting MAPK signalling pathway and inhibiting proliferation, metastasis, and angiogenesis as well as inducing apoptosis.

The PI3K/Akt pathway is an overactive intracellular signalling pathway in prostate cancer that is involved in the regulation of apoptosis, cell cycle progression, and cellular growth [28, 29]. However, suppression of Akt proteins by *Phyllanthus *was observed, and this suppression could induce apoptosis through the activation of the proapoptotic factors such as Bad, GSK3*β*, procaspase-9, and TRAIL/APO-2L (TNF-related apoptosis-inducing ligand) [15, 30]. The activation of Bax and GSK3*β* was detected in the treated PC-3 cells. Induction of apoptosis by *Phyllanthus *will be further implemented when the transcription factor cyclic AMP response element-binding protein (CREB), and the I*κ*B kinase (IKK), a positive regulator of NF*κ*B, are dephosphorylated by Akt protein, thus possibly leading to a reduction in the expression of genes with antiapoptotic activity [30, 31].

Glycogen synthase kinase 3-beta (GSK3*β*), cyclin-dependent kinase inhibitors (p21^CIP1/WAF1^) and Raf are the downstream targets of active Akt to regulate protein synthesis, glycogen metabolism, and cell cycle regulation. In a cancer cell, activated Akt will phosphorylate GSK3*β*, thus inhibiting its function in the degradation of *β*-catenin and c-myc. The stabilization of *β*-catenin is also regulated by dishevelled (DSH) through inhibition of GSK3*β* activity (Figure 5) [32]. All these allow *β*-catenin and c-myc activities by translocating into the nucleus and initiating expression of several genes to promote growth such as cyclin D in cell cycle. However, down-regulation of Akt and DSH proteins by *Phyllanthus* could cause activation of GSK3*β* and degradation of *β*-catenin and c-myc (Figures 3(b) and 3(c)). This could then reduce the production of cyclin D and thus arrest the cell cycle at a G1-phase in *Phyllanthus*-treated PC-3, as was observed in our previous study [8]. The dephosphorylation of Akt on p21^CIP1/WAF1^ could also inhibit Cdk4/cyclin D complex formation and yet again induce cell cycle arrest of PC-3 by *Phyllanthus* extracts [33].

In benign and malignant human prostate tissue, NF*κ*B is constrictively active and is believed to play an antiapoptotic role [14]. NF*κ*B induces the expression of the Inhibitors of apoptosis (IAPs) which inhibit apoptosis through direct suppression of effector caspases (caspases-3, -6, -7, and -9). In addition, the secretion and activation of matrix metalloproteinaises (MMP) by active NF*κ*B is well documented [34]. However, the down-regulation of NF*κ*Bp50 and p52 proteins in treated PC-3 cells was noted (Figure 3(d)) and this could inhibit proliferation, metastasis, and angiogenesis and induce programmed cell death through suppression of MMPs, VEGF, IL-8, p53, and antiapoptotic proteins (bcl-xl, cIAP) [35, 36].

The rapid uncontrolled proliferation of cancer cells usually outpaces new blood vessels generation, hence resulting in insufficient blood supply/oxygen to tumour tissues. In this condition, the cancer cells are forced to upregulate the expression of genes and enzymes which are involved in anaerobic glycolytic pathway as the main route of energy production, and this phenomenon is known as the Warburg effect [37]. The expression of hypoxia-inducible factor (HIF) is activated during low oxygen level. The active HIF will mediate activation of multiple genes involved in angiogenesis (e.g., VEGF), cell survival (e.g., IGF-1), and metastasis (e.g., LOX and PAI-1) and this drives tumour progression [38]. This metabolic adaptation in response to these alterations is believed to be associated with resistance to therapeutic agents [39].

In our study, *Phyllanthus* extracts were noted to inhibit the glycolytic pathway and energy production in prostate cancer cells by down-regulating HIF-1*α* protein. The deactivated HIF-1*α* protein will reduce the production VEGF and thus inhibit tumour angiogenesis and thereby decrease cancer progression (Figure 3(c)). In addition, several glycolytic enzymes were significantly downregulated in PC-3 after treatment with *Phyllanthus* extracts such as phosphoglycerate kinase, urocortin-3, alpha-enolase, GAPDH, fructose-bisphosphate aldolase, triosephosphate isomerase, and neuroglobin (Table 2, Group III). Other signalling pathways involved in the Warburg effect such as PI3 K/Akt and Ras-MAPK were also suppressed by *Phyllanthus *[39].

The network of protein-protein interaction in cancer cells plays an important role in the regulation of cellular function and biological processes [40] to ensure malignancy of cancer cells. Many of these vital proteins were found to be altered by *Phyllanthus* in PC-3. Among them, 3 were members of the keratin family (Table 2, Group 1). Differentiation-specific keratin has been known to play a role in epithelial-mesenchymal transition (EMT). EMT is a vital process that gives rise to cancer cell characteristics such as increased migratory capacity, invasiveness, and resistance to apoptosis during metastasis. Tumour aggressiveness has been correlated to keratin downregulation, despite its exact mechanism being unclear. This downregulation of keratin could be due to the expression of vimentin [41], as was also observed in our study, where the upover exregulation of keratin was accompanied by a decrease in vimentin following treatment by *Phyllanthus*.

In cell cytosol, Akt protein protects vimentin from caspase-induced proteolysis, and in *Phyllanthus*-treated cells, Akt was downregulated and hence a downregulation of vimentin (Table 2, Group I). The overexpression of vimentin is always correlated with tumour growth and invasion, where vimetin is believed to regulate various intracellular signalling and cell cycle control pathways [42]. This is done by stabilization of the ERK protein and allowing it to be translocated into the nucleus. Therefore, down-regulation of vimentin could prevent the translocation of ERK which means decreased prostate tumour growth, adhesion, and invasion as well as apoptosis induction.

Glutathione S-transferases (GSTs) are a family of enzymes that play an important role in redox homeostasis [43]. These enzymes are highly expressed in cancer cells and are believed to limit the efficacy of chemotherapeutic agents. This occurs via detoxification whereby the agents are conjugated with reduced glutathione, causing them to be more water-soluble and enhances the elimination of the agents [44, 45]. Therefore, the suppression of GSTs in treated PC-3 cells could possibly bypass detoxification of *Phyllanthus *extracts allowing them to be circulated to target cancer areas (Table 2, Group II).

In the *Phyllanthus*-treated cells, six differentially expressed proteins have been associated with intracellular signaling: protein Wnt-5a, proto-oncogene Wnt-3, Bcl-2-like protein 11, probable G-protein coupled receptor 179, putative Ras-related protein Rab-42, and GTPase HRas precursor (Table 2, Group II). Both Wnt-5a and proto-oncogene Wnt-3 proteins are involved in Wnt signalling pathway and their reduction could inhibit the Wnt signalling pathway. The Bcl-2-like protein 11 has similar antiapoptotic function as Bcl-2 and their reduction is believed to initiate apoptosis in PC-3 cells. The probable G-protein-coupled receptor 179 plays an important role to initiate signal transduction in cells through its down-stream target, GTPase HRas precursor. The reduced expression of probable G-protein-coupled receptor 179 in treated cells could have probably reduced the sensitivity of cancer cells to growth factors and inhibits the GTPase HRas precursor to initiate the signal transduction in PC-3 cells.

Mitochondrion, a vital component cell, is best known for its oxidative phosphorylation ability to produce energy source, ATP [46]. Voltage-dependent anion-selective channel protein 1 (VDAC) is a mitochondrial outer membrane protein that regulates ATP/ADP exchange and respiratory control [47]. VDAC has been shown to be proapoptotic by regulation of Bak and Bax [48] as well as activation of caspase-8 to induce extrinsic apoptosis pathway [49]. Thus, we believe that up-regulation of VDAC by *Phyllanthus* extracts was able to initiate apoptotic cell death in PC-3 cells (Table 2, Group IV). In addition, down-regulation of calumenin, calreticulin, heat shock proteins, 78 kDa glucose-regulated protein, and mitochondrial inner membrane organizing system protein 1 (MINOS1) are believed to be attributed to alteration in intracellular calcium level in PC-3 cells, thus triggering apoptotic cell death (Table 2, Group IV) [50–53].

In conclusion, this study has provided a comprehensive perspective on how *Phyllanthus* could have induced anticancer properties in prostate cancer cells. Overall, cumulative results from experimental and predictive studies suggest that *Phyllanthus* exerts its antiproliferative and apoptotic effects through suppression of MAPKs, PI3K/Akt, Wnt, Myc/Max, Hypoxia, and NF*κ*B signalling cascade *in vitro *(Figure 5). Further *in vivo* studies with *Phyllanthus *either alone or in conjunction with existing chemotherapeutic drugs are needed to demonstrate the potential application of *Phyllanthus *for the treatment of prostate and other cancers.

## Figures and Tables

**Table 1:** Treatment of PC-3 cells at different IC_50_ values of *Phyllanthus* extracts [8].

*Phyllanthus * species	Extracts	IC_50_ ± SEM (*μ*g/mL)
		PC-3
*P. * *amarus (PA) *	Aqueous (H_2_O)	178.3 ± 2.8
	Methanolic (MeOH)	84.3 ± 1.1
*P. * *niruri (PN) *	Aqueous (H_2_0)	155.0 ± 1.2
	Methanolic (MeOH)	117.7 ± 2.1
*P. * *urinaria (PU) *	Aqueous (H_2_O)	155.7 ± 2.1
	Methanolic (MeOH)	54.2 ± 2.1
*P. * *watsonii (PW) *	Aqueous (H_2_O)	156.7 ± 2.4
	Methanolic (MeOH)	100.5 ± 1.2

**Table 2:** Fold changes of differential proteins in aqueous- and methanolic-treated PC-3 cells. Symbols “+” indicate upregulation and “−” indicate downregulation. PA: *P.* *amarus*; PN: *P.* *niruri*; PU: *P.* *urinaria* and PW: *P.* *watsonii*.

No.	UniProtKB/Swiss-Prot (accession number)	Protein	*Phyllanthus *							
			Aqueous extract				Methanolic extract			
			PA	PN	PU	PW	PA	PN	PU	PW
I	Cell adhesion, migration, invasion, and metastasis and angiogenesis									

1	P98172	Ephrin-B1	−1.53	−1.45	−1.21	−1.41	−2.14	−2.21	−2.01	−2.31
2	P05787	Keratin, type II cytoskeletal 8	1.45	1.32	1.38	1.21	1.86	1.74	1.92	1.62
3	P63261	Actin, cytoplasmic 2	−1.43	−1.53	−1.21	−1.53	−1.74	−1.79	−1.72	−1.53
4	Q8NDI1	EH domain-binding protein 1	−1.32	−1.32	−1.21	−1.13	−1.42	−1.32	−1.43	−1.43
5	P04792	Heat shock protein beta-1	−1.43	−1.32	−1.54	−1.54	−1.42	−1.43	−1.32	−1.25
6	P35527	Keratin, type I cytoskeletal 9	1.38	1.32	1.53	1.42	1.64	1.34	1.35	1.37
7	Q9BYR8	Keratin-associated protein 3-1	1.53	1.32	1.47	1.64	1.47	1.53	1.42	1.46
8	P08670	Vimentin	−1.43	−1.32	−1.32	−1.11	−1.25	−1.43	−1.32	−1.42
9	Q9NY65	Tubulin alpha-8 chain	−1.53	−1.43	−1.48	−1.35	−1.40	−1.43	−1.42	−1.73
10	Q9Y316	Protein MEMO1	−1.43	−1.42	−1.24	−1.32	−1.63	−1.32	−1.42	−1.43

II	Proliferation, cell cycle, and apoptosis									

11	Q92466	DNA damage-binding protein 2	−1.42	−1.32	−1.43	−1.41	−1.42	−1.37	−1.43	−1.32
12	Q7RTU1	Transcription factor 23	−1.39	−1.33	−1.42	−1.43	−1.53	−1.63	−1.58	−1.42
13	Q9UQ80	Proliferation-associated protein 2G4	−1.37	−1.57	−1.42	−1.47	−1.63	−1.45	−1.53	−1.43
14	P62993	Growth factor receptor-bound protein 2	−1.42	−1.52	−1.43	−1.32	−1.42	−1.42	−1.43	−1.64
15	O60565	Gremlin-1	−1.43	−1.43	−1.33	−1.43	−1.56	−1.43	−1.54	−1.44
16	P27348	14-3-3 protein theta	−1.32	−1.43	−1.53	−1.42	−1.42	−1.21	−1.32	−1.42
17	P61981	14-3-3 protein gamma	−1.32	−1.32	−1.43	−1.35	−1.32	−1.42	−1.22	−1.32
18	P04083	Annexin A1	−1.52	−1.54	−1.42	−1.43	−1.64	−1.43	−1.43	−1.43
19	Q96LY2	Coiled-coil domain-containing protein 74B	−1.47	−1.34	−1.47	−1.46	−1.33	−1.33	−1.36	−1.57
20	P31943	Heterogeneous nuclear ribonucleoprotein H	−1.47	−1.33	−1.35	−1.21	−1.44	−1.56	−1.43	−1.53
21	P09211	Glutathione S-transferase P	−1.96	−1.84	−1.77	−2.05	−1.89	−1.87	−2.01	−1.97
22	P41221	Protein Wnt-5a	−1.75	−1.87	−1.87	−1.92	−1.74	−1.84	−1.85	−2.01
23	Q9H0R3	Transmembrane protein 222	−1.57	−1.52	−1.57	−1.42	−1.47	−1.62	−1.57	−1.46
24	Q5MJ09	Sperm protein associated with the nucleus on the X chromosome N3	−1.45	−1.57	−1.57	−1.57	−1.34	−1.45	−1.32	−1.46
25	P46063	ATP-dependent DNA helicase Q1	−1.57	−1.57	−1.36	−1.46	−1.56	−1.47	−1.46	−1.57
26	P09382	Galectin-1	−1.25	−1.46	−1.57	−1.47	−1.46	−1.33	−1.46	−1.46
27	P04792	Heat shock protein beta-1	−1.37	−1.47	−1.47	−1.46	−1.56	−1.46	−1.34	−1.47
28	P78417	Glutathione transferase omega-1	−1.46	−1.46	−1.47	−1.47	−1.57	−1.54	−1.68	−1.57
29	Q06830	Peroxiredoxin-1	−1.57	−1.42	−1.29	−1.33	−1.48	−1.62	−1.54	−1.67
30	P30048	Thioredoxin-dependent peroxide reductase, mitochondrial	−1.45	−1.52	−1.12	−1.64	−1.58	−1.44	−1.57	−1.57
31	Q9Y230	RuvB-like 2	−1.46	−1.47	−1.38	−1.32	−1.75	−1.57	−1.64	−1.57
32	P50453	Serpin B9	−1.46	−1.50	−1.36	−1.38	−1.58	−1.67	−1.76	−1.63
33	Q8ND25	E3 ubiquitin-protein ligase ZNRF1	−1.56	−1.43	−1.47	−1.47	−1.58	−1.67	−1.82	−1.54
34	Q6PRD1	Probable G-protein-coupled receptor 179	−1.45	−1.43	−1.47	−1.56	−1.36	−1.37	−1.54	−1.58
35	O43521	Bcl-2-like protein 11	−1.66	−1.52	−1.42	−1.56	−1.57	−1.73	−1.65	1.56
36	P56703	Proto-oncogene Wnt-3 precursor	−2.18	−1.92	−2.06	−2.34	−1.79	−1.97	1.83	−2.11
37	Q8N4Z0	Putative Ras-related protein Rab-42	−2.11	−2.03	−1.79	−1.98	−1.96	−2.1	−2.15	−1.94
38	P01112	GTPase HRas precursor	−2.12	−1.99	−1.83	−1.74	−1.85	−2.03	−2.05	−1.92

III	Glycogenesis and glycolysis									

39	Q969E3	Urocortin-3	−1.37	−1.42	−1.37	−1.46	−1.65	−1.73	1.67	−1.47
40	P00558	Phosphoglycerate kinase 1	−2.01	−2.12	−2.22	−1.93	−1.56	−1.37	−1.57	−1.36
41	P06733	Alpha-enolase	−1.42	−1.38	−1.37	−1.36	−1.58	−1.57	−1.65	−1.57
42	P04406	Glyceraldehyde-3-phosphate dehydrogenase	−1.92	−1.88	−1.92	−1.83	−1.78	−1.36	−1.36	−1.56
43	P04075	Fructose-bisphosphate aldolase A	−1.63	−1.42	−1.39	−1.36	−1.48	−1.53	−1.52	−1.57
43	P60174	Triosephospate isomerase	−1.47	−1.46	−1.47	−1.76	−1.48	−1.53	−1.49	−1.49
44	Q9NPG2	Neuroglobin	−1.47	−1.47	−1.58	−1.47	−1.56	−1.63	−1.74	−1.74

IV	Protein synthesis and energy metabolism									

45	Q4U2R6	39S ribosomal protein L51	−1.37	−1.47	−1.36	−1.53	−1.64	−1.74	−1.65	−1.67
46	Q93088	Betaine-homocysteine S-methyltransferase 1	−1.46	−1.47	−1.46	−1.48	−1.67	−1.48	−1.58	−1.57
47	P50583	Bis(5′-nucleosyl)-tetraphosphatase [asymmetrical]	−1.45	−1.57	−1.30	−1.42	−1.39	−1.47	−1.67	−1.87
48	Q96A11	Galactose-3-O-sulfotransferase 3	−1.69	−1.74	−1.67	−1.85	−1.93	−1.74	−1.82	−1.89
49	Q9Y274	Type 2 lactosamine alpha-2,3-sialyltransferase	−1.28	−1.52	−1.58	−1.44	−1.68	−1.64	−1.58	−1.57
50	O43852	Calumenin	−1.53	−1.47	−1.47	−1.42	−1.57	−1.65	−1.56	−1.53
51	P13667	Protein disulfide-isomerase A4	−1.47	−1.41	−1.44	−1.45	−1.58	−1.64	−1.45	−1.57
52	P27797	Calreticulin	−1.54	−1.32	−1.47	−1.48	−1.45	−1.53	−1.57	−1.64
53	P07900	Heat shock protein HSP 90-alpha	−1.64	−1.32	−1.34	−1.43	−1.55	−1.42	−1.32	−1.53
54	P12235	ADP/ATP translocase 1	−1.46	−1.38	−1.45	−1.35	−1.58	−1.68	−1.67	−1.47
55	P52209	6-phosphogluconate dehydrogenase, decarboxylating	−1.46	−1.49	−1.54	−1.45	−1.57	−1.52	−1.48	−1.56
56	Q9Y478	5′-AMP-activated protein kinase subunit beta-1	−1.38	−1.46	−1.47	−1.47	−1.67	−1.75	−1.47	−1.57
57	P11021	78 kDa glucose-regulated protein	−1.46	−1.46	−1.47	−1.57	−1.57	−1.54	−1.57	−1.84
58	Q9UI09	NADH dehydrogenase [ubiquinone] 1 alpha subcomplex subunit 12	−1.42	−1.36	−1.33	−1.33	−1.57	−1.73	−1.57	−1.46
59	P30101	Protein disulfide-isomerase A3	−1.20	−1.47	−1.37	−1.21	−1.57	−1.63	−1.56	−1.57
60	P49411	Elongation factor Tu, mitochondrial	−1.48	−1.45	−1.57	−1.33	−1.38	−1.54	−1.68	−1.47
61	Q5TGZ0	Mitochondrial inner membrane organizing system protein 1	−1.47	−1.46	−1.47	−1.49	−1.57	−1.47	−1.67	−1.65
62	P19404	NADH dehydrogenase [ubiquinone] flavoprotein 2, mitochondrial	−1.21	−1.32	−1.33	−1.43	−1.57	−1.57	−1.57	−1.74
63	Q9H0F7	ADP-ribosylation factor-like protein 6	−1.76	−1.50	−1.57	−1.54	−1.56	−1.54	−1.49	−1.70
64	O75436	Vacuolar protein sorting-associated protein 26A	−1.46	−1.54	−1.63	−1.76	−1.47	−1.38	−1.57	−1.57
65	Q9NXV2	BTB/POZ domain-containing protein KCTD5	−1.47	−1.47	−1.33	−1.50	−1.58	−1.65	−1.67	−1.66
66	O00429	Dynamin-1-like protein	−1.47	−1.63	−1.47	−1.57	−1.56	−1.57	−1.67	−1.73
67	P40261	Nicotinamide N-methyltransferase	−1.29	−1.22	−1.34	−1.28	−1.45	−1.48	−1.83	−1.42
68	P49720	Proteasome subunit beta type-3	−1.53	−1.22	−1.34	−1.47	−1.58	−1.53	−1.67	−1.57
69	Q6IQ16	Speckle-type POZ protein-like	−1.42	−1.67	−1.47	−1.47	−1.63	−1.64	−1.68	−1.47
70	P21796	Voltage-dependent anion-selective channel protein 1	1.57	1.63	1.52	1.79	1.67	1.84	1.77	1.76
71	Q9Y3D8	Adenylate kinase isoenzyme 6	−1.51	−1.33	−1.43	−1.49	−1.46	−1.64	−1.74	−1.76
72	Q14152	Eukaryotic translation initiation factor 3 subunit 12	−1.40	−1.37	−1.39	−1.49	−1.53	−1.67	−1.63	−1.53
